# Bis(μ-biphenyl-2,2′-dicarboxyl­ato)bis­[(2,2′-bipyridine)copper(II)]

**DOI:** 10.1107/S1600536809008976

**Published:** 2009-03-19

**Authors:** Zhe An, Rui-Hai Cui, Ru-Jin Zhou

**Affiliations:** a School of Chemistry and Life Science, Maoming University, Maoming 525000, People’s Republic of China; b School of Chemistry and Life Science, Harbin University, Harbin 150080, People’s Republic of China

## Abstract

The title compound, [Cu_2_(C_14_H_8_O_4_)_2_(C_10_H_8_N_2_)_2_], was obtained by solvothermal synthesis. The Cu^II^ atom is coordinated by one chelating 2,2′-bipyridine ligand and two carboxyl groups from different biphenyl-2,2′-dicarboxyl­ate ligands, leading to a distorted octahedral environment. Each carboxyl­ate group makes one short Cu—O bond [1.9608 (14) and 1.9701 (14) Å] and one longer Cu—O contact [2.4338 (17) and 2.5541 (17) Å] to each Cu^II^ atom. The biphenyl-2,2′-dicarboxyl­ate ligands bridge between Cu^II^ atoms, forming a dinuclear complex around a crystallographic inversion centre.

## Related literature

For complexes of biphenyl-2,2′-dicarboxylic acid, a good candidate for the construction of metal–organic frameworks, see: Rueff *et al.* (2003 ▶); Xu *et al.* (2006 ▶); An & Niu (2008 ▶).
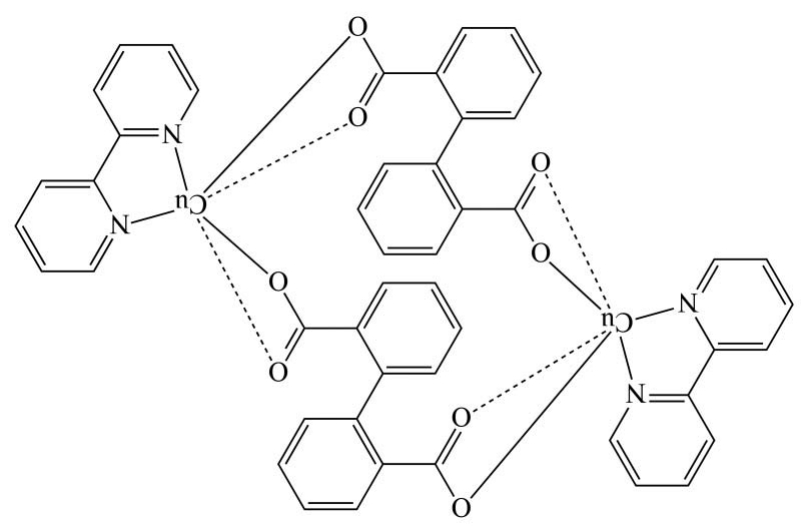


## Experimental

### 

#### Crystal data

[Cu_2_(C_14_H_8_O_4_)_2_(C_10_H_8_N_2_)_2_]
*M* *_r_* = 919.86
Monoclinic, 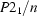

*a* = 11.220 (2) Å
*b* = 13.350 (3) Å
*c* = 13.400 (3) Å
β = 103.02 (3)°
*V* = 1955.5 (7) Å^3^
*Z* = 2
Mo *K*α radiation
μ = 1.15 mm^−1^
*T* = 296 K
0.12 × 0.10 × 0.08 mm

#### Data collection

Bruker APEXII CCD diffractometer
Absorption correction: multi-scan (*SADABS*; Sheldrick, 2003 ▶) *T*_min_ = 0.874, *T*_max_ = 0.913
10453 measured reflections
3644 independent reflections
3099 reflections with *I* > 2σ(*I*)
*R*_int_ = 0.019

#### Refinement

*R*[*F* ^2^ > 2σ(*F* ^2^)] = 0.027
*wR*(*F* ^2^) = 0.088
*S* = 1.00
3644 reflections
280 parameters
H-atom parameters not refined
Δρ_max_ = 0.27 e Å^−3^
Δρ_min_ = −0.29 e Å^−3^

### 

Data collection: *APEX2* (Bruker, 2004 ▶); cell refinement: *SAINT-Plus* (Bruker, 2001 ▶); data reduction: *SAINT-Plus*; program(s) used to solve structure: *SHELXS97* (Sheldrick, 2008 ▶); program(s) used to refine structure: *SHELXL97* (Sheldrick, 2008 ▶); molecular graphics: *SHELXTL* (Sheldrick, 2008 ▶); software used to prepare material for publication: *SHELXTL*.

## Supplementary Material

Crystal structure: contains datablocks I, global. DOI: 10.1107/S1600536809008976/bi2358sup1.cif

Structure factors: contains datablocks I. DOI: 10.1107/S1600536809008976/bi2358Isup2.hkl

Additional supplementary materials:  crystallographic information; 3D view; checkCIF report

## Figures and Tables

**Fig. 1.**
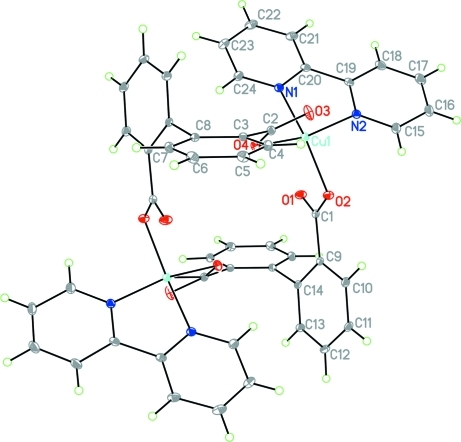
Molecular structure drawn with 30% probability displacement ellipsoids for the non-H atoms. Unlabelled atoms are related to labelled atoms by the symmetry code 1 - x, 1 - y, 1 - z.

## supplementary crystallographic information

### Comment

Biphenyl-2,2'-dicarboxylic acid (H_2_dpa) has been demonstrated to be a good candidate for the construction of metal-organic frameworks, and some complexes based on 2,2'-dpa have been reported (Rueff *et al.*, 2003; Xu *et al.*, 2006; An & Niu, 2008). In this paper, we report a new metal complex constructed from dpa, 2,2-bipyridine and copper(II) (Figure 1).

### Experimental

A mixture of Cu(CH_3_COO)_2_.H_2_O (1 mmol), biphenyl-2,2'-dicarboxylic acid (1 mmol), and 2,2'-bipyridine (1 mmol) in 20 ml methanol/water (1:1) were placed in a 25 ml Teflon-lined stainless steel autoclave and kept at 453 K for five days. Blue crystals were obtained after cooling to room temperature.

### Refinement

All H atoms were placed in calculated positions with C—H = 0.93 Å and refined as riding with *U*_iso_(H) = 1.2*U*_eq_(C).

### Crystal data

**Table:**

[Cu_2_(C_14_H_8_O_4_)_2_(C_10_H_8_N_2_)_2_]	*F*(000) = 940
*M**_r_* = 919.86	*D*_x_ = 1.562 Mg m^−^^3^
Monoclinic, *P*2_1_/*n*	Mo *K*α radiation, λ = 0.71073 Å
Hall symbol: -P 2yn	Cell parameters from 3644 reflections
*a* = 11.220 (2) Å	θ = 2.1–25.5°
*b* = 13.350 (3) Å	µ = 1.15 mm^−^^1^
*c* = 13.400 (3) Å	*T* = 296 K
β = 103.02 (3)°	Block, blue
*V* = 1955.5 (7) Å^3^	0.12 × 0.10 × 0.08 mm
*Z* = 2	

### Data collection

**Table:**

Bruker APEXII CCD diffractometer	3644 independent reflections
Radiation source: fine-focus sealed tube	3099 reflections with *I* > 2σ(*I*)
graphite	*R*_int_ = 0.019
φ and ω scans	θ_max_ = 25.5°, θ_min_ = 2.1°
Absorption correction: multi-scan (*SADABS*; Sheldrick, 2003)	*h* = −13→13
*T*_min_ = 0.874, *T*_max_ = 0.913	*k* = −16→10
10453 measured reflections	*l* = −16→14

### Refinement

**Table:**

Refinement on *F*^2^	Primary atom site location: structure-invariant direct methods
Least-squares matrix: full	Secondary atom site location: difference Fourier map
*R*[*F*^2^ > 2σ(*F*^2^)] = 0.027	Hydrogen site location: inferred from neighbouring sites
*wR*(*F*^2^) = 0.088	H-atom parameters not refined
*S* = 1.00	*w* = 1/[σ^2^(*F*_o_^2^) + (0.063*P*)^2^ + 0.1*P*] where *P* = (*F*_o_^2^ + 2*F*_c_^2^)/3
3644 reflections	(Δ/σ)_max_ = 0.031
280 parameters	Δρ_max_ = 0.27 e Å^−^^3^
0 restraints	Δρ_min_ = −0.29 e Å^−^^3^

### Special details

**Table:**

Geometry. All e.s.d.'s (except the e.s.d. in the dihedral angle between two l.s. planes) are estimated using the full covariance matrix. The cell e.s.d.'s are taken into account individually in the estimation of e.s.d.'s in distances, angles and torsion angles; correlations between e.s.d.'s in cell parameters are only used when they are defined by crystal symmetry. An approximate (isotropic) treatment of cell e.s.d.'s is used for estimating e.s.d.'s involving l.s. planes.
Refinement. Refinement of *F*^2^ against ALL reflections. The weighted *R*-factor *wR* and goodness of fit *S* are based on *F*^2^, conventional *R*-factors *R* are based on *F*, with *F* set to zero for negative *F*^2^. The threshold expression of *F*^2^ > σ(*F*^2^) is used only for calculating *R*-factors(gt) *etc*. and is not relevant to the choice of reflections for refinement. *R*-factors based on *F*^2^ are statistically about twice as large as those based on *F*, and *R*- factors based on ALL data will be even larger.

### Fractional atomic coordinates and isotropic or equivalent isotropic displacement parameters (Å^2^)

**Table:**

	*x*	*y*	*z*	*U*_iso_*/*U*_eq_	
C1	0.52301 (18)	0.68894 (15)	0.42300 (15)	0.0295 (4)	
C2	0.28746 (17)	0.57752 (15)	0.60591 (15)	0.0293 (4)	
C3	0.30695 (16)	0.51522 (15)	0.70154 (14)	0.0270 (4)	
C4	0.33117 (18)	0.56634 (16)	0.79532 (15)	0.0341 (5)	
H4	0.3274	0.6359	0.7960	0.041*	
C5	0.36046 (19)	0.51453 (18)	0.88649 (15)	0.0393 (5)	
H5	0.3786	0.5489	0.9484	0.047*	
C6	0.3627 (2)	0.41049 (18)	0.88508 (16)	0.0404 (5)	
H6	0.3821	0.3752	0.9464	0.048*	
C7	0.33624 (19)	0.35926 (16)	0.79356 (16)	0.0344 (5)	
H7	0.3370	0.2896	0.7939	0.041*	
C8	0.30828 (16)	0.41025 (15)	0.70031 (14)	0.0268 (4)	
C9	0.66065 (16)	0.69929 (14)	0.45025 (14)	0.0270 (4)	
C10	0.71452 (18)	0.75479 (16)	0.53682 (16)	0.0358 (5)	
H10	0.6646	0.7884	0.5725	0.043*	
C11	0.83990 (19)	0.76120 (16)	0.57103 (17)	0.0388 (5)	
H11	0.8741	0.7992	0.6285	0.047*	
C12	0.91368 (18)	0.71022 (17)	0.51856 (17)	0.0393 (5)	
H12	0.9983	0.7130	0.5410	0.047*	
C13	0.86141 (19)	0.65506 (15)	0.43273 (17)	0.0339 (5)	
H13	0.9120	0.6208	0.3982	0.041*	
C14	0.73499 (18)	0.64916 (13)	0.39621 (15)	0.0270 (4)	
C15	0.2751 (2)	0.87802 (18)	0.39294 (19)	0.0426 (5)	
H15	0.3426	0.8868	0.4470	0.051*	
C16	0.2221 (2)	0.96089 (18)	0.3405 (2)	0.0522 (7)	
H16	0.2509	1.0248	0.3605	0.063*	
C17	0.1257 (2)	0.9477 (2)	0.2579 (2)	0.0557 (7)	
H17	0.0896	1.0026	0.2202	0.067*	
C18	0.0830 (2)	0.85266 (19)	0.2316 (2)	0.0463 (6)	
H18	0.0188	0.8425	0.1751	0.056*	
C19	0.13647 (18)	0.77206 (16)	0.29011 (15)	0.0320 (5)	
C20	0.09346 (18)	0.66758 (16)	0.27505 (16)	0.0322 (5)	
C21	−0.0050 (2)	0.6365 (2)	0.19990 (18)	0.0442 (6)	
H21	−0.0477	0.6818	0.1522	0.053*	
C22	−0.0390 (2)	0.5368 (2)	0.19696 (18)	0.0511 (7)	
H22	−0.1050	0.5145	0.1468	0.061*	
C23	0.0242 (2)	0.4706 (2)	0.26769 (19)	0.0481 (6)	
H23	0.0014	0.4036	0.2664	0.058*	
C24	0.1223 (2)	0.50567 (17)	0.34091 (17)	0.0390 (5)	
H24	0.1658	0.4612	0.3890	0.047*	
Cu1	0.30127 (2)	0.660694 (17)	0.440302 (17)	0.033 (2)	
N1	0.15643 (14)	0.60194 (13)	0.34444 (12)	0.0304 (4)	
N2	0.23357 (15)	0.78535 (13)	0.36946 (12)	0.0309 (4)	
O1	0.46909 (13)	0.65650 (12)	0.33944 (13)	0.0462 (4)	
O2	0.46709 (12)	0.71453 (11)	0.49257 (10)	0.0356 (3)	
O3	0.23951 (18)	0.66102 (11)	0.60315 (13)	0.0510 (5)	
O4	0.32843 (13)	0.54377 (10)	0.53117 (10)	0.0336 (3)	

### Atomic displacement parameters (Å^2^)

**Table:**

	*U*^11^	*U*^22^	*U*^33^	*U*^12^	*U*^13^	*U*^23^
C1	0.0289 (10)	0.0228 (10)	0.0359 (11)	−0.0009 (8)	0.0052 (9)	−0.0015 (8)
C2	0.0303 (10)	0.0261 (11)	0.0293 (10)	−0.0008 (9)	0.0020 (8)	−0.0012 (8)
C3	0.0237 (9)	0.0287 (11)	0.0276 (10)	0.0006 (8)	0.0037 (8)	−0.0014 (8)
C4	0.0347 (11)	0.0317 (11)	0.0353 (11)	−0.0009 (9)	0.0066 (9)	−0.0056 (9)
C5	0.0389 (12)	0.0508 (14)	0.0255 (11)	0.0029 (10)	0.0017 (9)	−0.0075 (9)
C6	0.0429 (12)	0.0495 (14)	0.0281 (11)	0.0081 (11)	0.0064 (9)	0.0077 (10)
C7	0.0384 (12)	0.0307 (11)	0.0345 (12)	0.0044 (9)	0.0092 (9)	0.0057 (9)
C8	0.0242 (9)	0.0282 (11)	0.0285 (10)	0.0001 (8)	0.0070 (8)	0.0000 (8)
C9	0.0272 (10)	0.0219 (10)	0.0306 (10)	−0.0026 (8)	0.0037 (8)	−0.0006 (8)
C10	0.0384 (12)	0.0289 (11)	0.0410 (12)	−0.0041 (9)	0.0112 (10)	−0.0089 (9)
C11	0.0411 (12)	0.0324 (12)	0.0389 (12)	−0.0111 (10)	0.0006 (10)	−0.0098 (9)
C12	0.0273 (10)	0.0399 (13)	0.0474 (13)	−0.0093 (9)	0.0017 (9)	−0.0005 (10)
C13	0.0294 (11)	0.0344 (12)	0.0386 (12)	−0.0011 (9)	0.0094 (9)	−0.0025 (9)
C14	0.0285 (10)	0.0207 (10)	0.0302 (11)	−0.0030 (8)	0.0035 (8)	0.0037 (8)
C15	0.0469 (13)	0.0349 (13)	0.0462 (13)	0.0000 (11)	0.0107 (11)	0.0032 (10)
C16	0.0572 (16)	0.0295 (13)	0.0745 (18)	0.0032 (11)	0.0246 (14)	0.0086 (12)
C17	0.0516 (15)	0.0475 (16)	0.0698 (18)	0.0163 (12)	0.0177 (13)	0.0268 (13)
C18	0.0385 (13)	0.0495 (16)	0.0488 (15)	0.0134 (11)	0.0053 (11)	0.0158 (11)
C19	0.0284 (10)	0.0400 (12)	0.0292 (10)	0.0078 (9)	0.0095 (8)	0.0072 (9)
C20	0.0260 (10)	0.0433 (13)	0.0281 (11)	0.0036 (9)	0.0077 (8)	0.0052 (9)
C21	0.0310 (11)	0.0635 (17)	0.0344 (12)	−0.0044 (11)	−0.0006 (9)	0.0078 (11)
C22	0.0376 (13)	0.0725 (19)	0.0395 (14)	−0.0156 (12)	0.0010 (11)	−0.0042 (12)
C23	0.0442 (13)	0.0493 (15)	0.0501 (15)	−0.0174 (11)	0.0089 (11)	−0.0039 (12)
C24	0.0381 (11)	0.0380 (13)	0.0391 (12)	−0.0041 (10)	0.0051 (10)	0.0015 (10)
Cu1	0.045 (5)	0.025 (4)	0.028 (4)	−0.004 (4)	0.002 (4)	0.002 (3)
N1	0.0279 (8)	0.0338 (10)	0.0284 (9)	−0.0003 (7)	0.0042 (7)	0.0018 (7)
N2	0.0308 (9)	0.0305 (10)	0.0315 (9)	0.0017 (7)	0.0069 (7)	0.0038 (7)
O1	0.0303 (8)	0.0595 (11)	0.0457 (10)	−0.0038 (7)	0.0022 (7)	−0.0248 (8)
O2	0.0284 (7)	0.0461 (10)	0.0318 (8)	−0.0007 (6)	0.0058 (6)	−0.0052 (6)
O3	0.0790 (13)	0.0343 (10)	0.0429 (10)	0.0232 (8)	0.0207 (9)	0.0070 (7)
O4	0.0423 (8)	0.0315 (8)	0.0269 (7)	0.0067 (6)	0.0078 (6)	0.0043 (6)

### Geometric parameters (Å, °)

**Table:**

Cu1—O1	2.5541 (17)	C11—H11	0.930
Cu1—O2	1.9701 (14)	C12—C13	1.380 (3)
Cu1—O3	2.4338 (17)	C12—H12	0.930
Cu1—O4	1.9608 (14)	C13—C14	1.395 (3)
Cu1—N1	1.9914 (17)	C13—H13	0.930
Cu1—N2	1.9806 (17)	C14—C8^i^	1.502 (3)
C1—O1	1.226 (2)	C15—N2	1.334 (3)
C1—O2	1.282 (2)	C15—C16	1.372 (3)
C1—C9	1.511 (3)	C15—H15	0.930
C2—O3	1.235 (2)	C16—C17	1.373 (4)
C2—O4	1.275 (2)	C16—H16	0.930
C2—C3	1.502 (3)	C17—C18	1.374 (4)
C3—C8	1.402 (3)	C17—H17	0.930
C3—C4	1.402 (3)	C18—C19	1.386 (3)
C4—C5	1.378 (3)	C18—H18	0.930
C4—H4	0.930	C19—N2	1.352 (3)
C5—C6	1.389 (3)	C19—C20	1.475 (3)
C5—H5	0.930	C20—N1	1.355 (3)
C6—C7	1.377 (3)	C20—C21	1.381 (3)
C6—H6	0.930	C21—C22	1.383 (4)
C7—C8	1.395 (3)	C21—H21	0.930
C7—H7	0.930	C22—C23	1.370 (4)
C8—C14^i^	1.502 (3)	C22—H22	0.930
C9—C14	1.393 (3)	C23—C24	1.381 (3)
C9—C10	1.394 (3)	C23—H23	0.9300
C10—C11	1.381 (3)	C24—N1	1.339 (3)
C10—H10	0.930	C24—H24	0.930
C11—C12	1.380 (3)		
			
O1—C1—O2	122.48 (18)	C17—C16—H16	120.6
O1—C1—C9	121.35 (18)	C15—C16—H16	120.6
O2—C1—C9	116.17 (17)	C16—C17—C18	119.4 (2)
O3—C2—O4	121.83 (18)	C16—C17—H17	120.3
O3—C2—C3	120.29 (18)	C18—C17—H17	120.3
O4—C2—C3	117.75 (17)	C19—C18—C17	119.4 (2)
C8—C3—C4	119.79 (18)	C19—C18—H18	120.3
C8—C3—C2	122.93 (17)	C17—C18—H18	120.3
C4—C3—C2	117.19 (18)	N2—C19—C18	120.8 (2)
C5—C4—C3	120.7 (2)	N2—C19—C20	114.37 (17)
C5—C4—H4	119.7	C18—C19—C20	124.8 (2)
C3—C4—H4	119.7	N1—C20—C21	121.0 (2)
C4—C5—C6	119.4 (2)	N1—C20—C19	114.45 (18)
C4—C5—H5	120.3	C21—C20—C19	124.6 (2)
C6—C5—H5	120.3	C20—C21—C22	118.7 (2)
C7—C6—C5	120.52 (19)	C20—C21—H21	120.7
C7—C6—H6	119.7	C22—C21—H21	120.7
C5—C6—H6	119.7	C23—C22—C21	120.3 (2)
C6—C7—C8	121.0 (2)	C23—C22—H22	119.9
C6—C7—H7	119.5	C21—C22—H22	119.9
C8—C7—H7	119.5	C22—C23—C24	118.6 (2)
C7—C8—C3	118.56 (18)	C22—C23—H23	120.7
C7—C8—C14^i^	118.53 (18)	C24—C23—H23	120.7
C3—C8—C14^i^	122.41 (17)	N1—C24—C23	121.8 (2)
C14—C9—C10	119.32 (18)	N1—C24—H24	119.1
C14—C9—C1	121.98 (17)	C23—C24—H24	119.1
C10—C9—C1	118.52 (17)	O4—Cu1—O2	93.87 (6)
C11—C10—C9	121.83 (19)	O4—Cu1—N2	162.88 (6)
C11—C10—H10	119.1	O2—Cu1—N2	95.36 (7)
C9—C10—H10	119.1	O4—Cu1—N1	94.42 (7)
C12—C11—C10	118.92 (19)	O2—Cu1—N1	160.23 (6)
C12—C11—H11	120.5	N2—Cu1—N1	81.52 (7)
C10—C11—H11	120.5	O4—Cu1—O3	58.70 (5)
C11—C12—C13	119.77 (19)	O2—Cu1—O3	96.74 (7)
C11—C12—H12	120.1	N2—Cu1—O3	105.80 (6)
C13—C12—H12	120.1	N1—Cu1—O3	102.90 (7)
C14—C13—C12	122.0 (2)	C24—N1—C20	119.72 (18)
C14—C13—H13	119.0	C24—N1—Cu1	125.89 (14)
C12—C13—H13	119.0	C20—N1—Cu1	114.30 (14)
C13—C14—C9	118.12 (18)	C15—N2—C19	118.94 (18)
C13—C14—C8^i^	115.98 (17)	C15—N2—Cu1	126.17 (15)
C9—C14—C8^i^	125.89 (17)	C19—N2—Cu1	114.89 (14)
N2—C15—C16	122.6 (2)	C1—O2—Cu1	102.82 (12)
N2—C15—H15	118.7	C2—O3—Cu1	79.35 (12)
C16—C15—H15	118.7	C2—O4—Cu1	99.98 (12)
C17—C16—C15	118.8 (2)		

Symmetry codes: (i) −*x*+1, −*y*+1, −*z*+1.

## References

[bb1] An, Z. & Niu, X.-C. (2008). *Acta Cryst.* E**64**, m1556. 10.1107/S1600536808037288 PMC2959843 21581165

[bb2] Bruker (2001). *SAINT-Plus* Bruker AXS Inc., Madison, Wisconsin, USA.

[bb3] Bruker (2004). *APEX2* Bruker AXS Inc., Madison, Wisconsin, USA.

[bb4] Rueff, J.-M., Pillet, S., Bonaventure, G., Souhassou, M. & Rabu, P. (2003). *Eur. J. Inorg. Chem.* pp. 4173–4178.

[bb5] Sheldrick, G. M. (2003). *SADABS* University of Göttingen, Germany.

[bb6] Sheldrick, G. M. (2008). *Acta Cryst.* A**64**, 112–122. 10.1107/S0108767307043930 18156677

[bb7] Xu, X.-X., Lu, Y., Wang, E.-B., Ma, Y. & Bai, X.-L. (2006). *Cryst. Growth Des.* **6**, 2029–2035.

